# Retinal vessels modifications in acute and post-COVID-19

**DOI:** 10.1038/s41598-021-98873-1

**Published:** 2021-09-29

**Authors:** Alessandro Invernizzi, Marco Schiuma, Salvatore Parrulli, Alessandro Torre, Federico Zicarelli, Valeria Colombo, Sara Marini, Elena Villella, Alice Bertoni, Spinello Antinori, Giuliano Rizzardini, Massimo Galli, Luca Meroni, Andrea Giacomelli, Giovanni Staurenghi

**Affiliations:** 1 Eye Clinic, Luigi Sacco Hospital, ASST Fatebenefratelli-Sacco, University of Milan, Via G.B. Grassi 74, 20157 Milan, Italy; 2 Department of Biomedical and Clinical Sciences “L. Sacco”, Luigi Sacco Hospital, University of Milan, Milan, Italy; 3 The Discipline of Clinical Ophthalmology and Eye Health, Faculty of Medicine and Health, Save Sight Institute, Sydney Eye Hospital, The University of Sydney, 8 Macquarie Street, Sydney, NSW 2001 Australia; 4 Department of Infectious Diseases, ASST Fatebenefratelli-Sacco, Milan, Italy

**Keywords:** Eye manifestations, Retina

## Abstract

Coronavirus disease 2019 (COVID-19) is an infectious disease caused by SARS-CoV-2 primarily affecting the respiratory system which can damage vessels walls virtually in any body district. Changes affecting retinal vessels are a good marker for systemic vascular alterations. This study investigated retinal vessels during the acute phase of COVID-19 and after patients recovery. Fifty-nine eyes from 32 COVID-19 patients and 80 eyes from 53 unexposed subjects were included. Mean arteries diameter (MAD) and mean veins diameter (MVD) were assessed through semi-automatic analysis on fundus color photos at baseline and 6 months later in patients and subjects unexposed to the virus. At baseline MAD and MVD were significantly higher in COVID-19 patients compared to unexposed subjects (p < 0.0001). Both MAD and MVD significantly decreased in COVID-19 patients at follow-up (from 97.5 ± 10.9 to 92.2 ± 11.4 µm, p < 0.0001 and from 133.1 ± 19.3 to 124.6 ± 16.1 µm, p < 0.0001, respectively). Despite this reduction vessels diameter remained significantly higher in severe COVID-19 patients compared to unexposed subjects. Transient retinal vessels dilation could serve a biomarker for systemic inflammation while long-lasting alterations seen in severe COVID-19 likely reflect irreversible structural damage to the vessels walls and should be further investigated for their possible effects on tissues perfusion and function.

## Introduction

After its first outbreak in December 2019, Severe Acute Respiratory Syndrome-Coronavirus-2 (SARS-CoV-2) quickly spread across the globe turning into the first pandemic of the twenty-first century^1^. While many subjects affected by this virus are totally asymptomatic, about 40% of them develop symptoms and 10–20% of those requiring hospitalization suffer from respiratory distress and thromboembolic disorders leading to acute respiratory distress syndrome and, potentially, to death. The condition is named coronavirus diseases 2019 (COVID-19)^2^.

The eyes can be affected at many levels by SARS-CoV-2 as the virus has been isolated in the tears^3^, the conjunctiva^4^ and even the retina^5^. Although no disease-specific lesions have been described neither in the anterior nor in the posterior segment of the eye many authors have reported the occurrence of direct or indirect signs of retinal circulation alterations in patients with COVID-19 ranging from retinal haemorrhages and cotton wool spots^6^, retinal vein occlusions^7,8^ to a significant reduction in vascular density measured by optical coherence tomography angiography^9–12^.

Changes to retinal vessels can serve as a mirror for vascular alterations affecting the whole body. Recently, our group found that both retinal arteries and veins of patients with acute COVID-19 were significantly dilated compared to those of subjects unexposed to the virus. In addition, veins diameter correlated directly with the disease severity and inversely with the time from the symptoms onset^6^. Our findings unveiled this previously unknown effect of the disease on the retinal vasculature in vivo, but we could not determine whether they depended on a virus-induced damage to the vessels or they were a consequence of the massive inflammatory response characterizing COVID-19.

In this study we re-examined the same population 6 months later to assess whether the retinal vessels dilation that we had found during the acute phase of the disease regressed with time or the vascular changes persisted after the inflammation had worn off.

## Methods

During the first pandemic wave (May 2020) consecutive patients admitted to the Infectious Diseases Department of a tertiary referral centre in northern Italy (ASST-FBF-PO Luigi Sacco, University of Milan, Milan, Italy) diagnosed with acute COVID-19 (had a positive nasopharyngeal swab and had symptoms onset within 30 days before the fundus screening) and a population of subjects unexposed to the virus (subjects among hospital and university staff, who were asymptomatic during the previous month and tested negative for SARS-CoV-2 specific antibodies) were enrolled in the “ScrEening the Retina in Patients with COvid-19” (SERPICO-19) study^6^. The study was approved by the local ethic committee (Comitato Etico Milano Area 1—Protocol Number 2020/ST/088) and written informed consent was obtained from all participants. The study adhered to the tenants of the declaration of Helsinki.

This is the second report of the study describing follow-up findings. The results of the cross-sectional analysis as well as the inclusion criteria and the methods for the dataset acquisition at baseline have been extensively described in the first report^6^. For this reason only procedures and results relevant to the current analysis will be reported. In brief, in May 2020 fundus colour photos were collected in patients with acute COVID-19 and subjects unexposed to the virus and retinal features were evaluated and compared between the two groups. The mean diameter of retinal arteries (MAD) and veins (MVD) was also calculated with a semi-automatic approach and compared between COVID-19 patients and unexposed subjects. In addition, the vessels diameter was correlated with clinical parameters in the COVID-19 population.

### Baseline examination

Information regarding the age, sex, ethnicity, body mass index (BMI), smoking, alcohol consumption, presence of comorbidities (systemic hypertension, diabetes, dyslipidaemia, history of coronary disease/stroke, tuberculosis and HIV infection) were collected in all enrolled subjects. Comorbidities were considered as binary variables (YES/NO) without further characterization^6^.

Clinical information and laboratory parameters of COVID-19 patients were also collected. A Complete list can be found in the first report of the study^6^.

Patients were classified as having severe COVID-19 if they had any of the following: hypoxia (oxygen saturation ≤ 93 percent on room air or PaO2/FiO2 < 300 mmHg), tachypnoea (respiratory rate > 30 breaths per minute) or respiratory distress, more than 50 percent involvement of the lung parenchyma on chest imaging^6^. The remaining patients were classified as non-severe^13^.

### Follow-up examination

All subjects included in the first analysis that had given permission for being re-called were re-contacted by phone. Those who accepted to participate to this second part of the study were re-examined. The second examination was performed after 6 months (± 30 days) from the first fundus examination.

Blood samples were collected in patients with a previous COVID-19 history and the following parameters were investigated: haematocrit, white blood cells, neutrophils, lymphocytes and platelets count, prothrombin time (PT), partial thromboplastin time (PTT), Fibrinogen, D-Dimer, C reactive protein (CRP) and creatinine.

Subjects who had served as unexposed population at baseline were excluded from the follow-up if they developed symptoms and tested positive at nasopharyngeal swab for SARS-CoV-2. Asymptomatic infection was excluded by means of a serological assessment performed before the follow-up visit (DiaSorin LIAISON CLIA S1-S2 IgG, SALUGGIA (Vercelli), Italy). Only those with negative IgG were considered as unexposed during the follow-up period and included in the analysis.

### Retinal images acquisition

At baseline and follow-up visit all subjects underwent pupil dilation of both eyes using mydriatic drops (Tropicamide 1%) 15 min prior to the acquisition of retinal images. Two sets of fundus photos, one for each eye, were acquired in all subjects with the Digital Retinography System (DRS) fundus camera (CenterVue, Padua, Italy). Each set was constituted by four photos, with a 45° × 40° field of view each and a resolution of 48 pixels/degree, two centred on the macular area and two on the optic nerve head^6^.

### Images analysis

Fundus images of both eyes collected at baseline and follow-up examination were examined by two retinal specialists (FZ and SP) to evaluate their quality. If the fundus details were not visible due to acquisition artefacts or media opacities in more than 2/4 photos the eye was not included in the analysis, if both eyes were graded “unreadable” the subject was excluded. The same graders assessed the images for the presence of retinal abnormalities. In case of disagreement, a third senior retinal specialist (AI) was asked to evaluate the image^6^.

Fundus colour photos collected at follow-up were assessed for the presence of retinal alterations including retinal haemorrhages, cotton wool spots, drusen, dilated veins and tortuous vessels by two retinal specialists. In case of disagreement, a third senior retinal specialist (AI) was asked to evaluate the image. The results were compared to those from the same assessment performed 6 months earlier on the images collected at baseline.

The fundus colour photo centred onto the optic nerve was used to assess the MAD and MVD using a semi-automatic approach. Retinal images were acquired and processed using the Automated Retinal Image Analyzer (ARIA, V1-09-12-11), an opensource software developed on the MATLAB platform (MATLAB R2020a—update 1 (9.8.0.1359463))^6,14^. Using this software we calculated the diameter of the four main arteries and veins between 0·5 and 1 disk diameters from the optic disk margin^15,16^ and obtained the MAD and MVD by averaging the arterial and venous values respectively^6^.

### Statistical analysis

Descriptive statistics for continuous variables included the mean, standard deviation (SD), and ranges where appropriate. The prevalence of demographic qualitative variables was reported in percentage^6^. Comparisons between baseline and follow-up findings within groups were performed by means of McNemar test for binary variables. Difference in MAD and MVD from baseline to follow-up within groups was analysed by means of a linear mixed effect model for repeated measurements accounting for nesting of the eyes within subjects.

The effect of severe and non-severe COVID-19 on MAD and MVD was tested at baseline and follow-up using mixed multiple linear regression analyses accounting for nesting of the eyes within subjects, considering unexposed subjects as reference and including a priori arbitrary chose confounders defined as factors known to have a possible effect on retinal vessels (age, sex, ethnicity, BMI, systemic hypertension, diabetes, smoking, alcohol consumption, dyslipidaemia, history of coronary disease/stroke) as covariates^6^. This allowed to compare COVID-19 patients and unexposed subjects accounting for the different distribution of covariates in the two populations. Bonferroni correction was applied for multiple comparisons between groups. The statistical analyses were run on R Studio (Version 1.1.383, R Project, www.r-project.org). p values < 0·05 were considered statistically significant.

## Results

Out of the 54 COVID-19 patients and the 133 unexposed subjects included in the cross-sectional analysis (SERPICO-19 Report 1) 33 (61.1%) and 64 (48.1%) respectively agreed to join the follow-up study. Eleven out of the 64 subjects enrolled as unexposed during the cross-sectional analysis, who agreed to join the current analysis had a positive serology for SARS-CoV-2 at follow-up examination and were consequently excluded from the follow-up analysis. One COVID-19 patient was excluded for bad quality images at follow-up. For the detailed reasons for subjects drop-out see the diagram reported in Supplement Material 1.

Fifty-nine eyes from 32 COVID-19 patients and 80 eyes from 53 unexposed subjects had gradable fundus images both at baseline and follow-up and were included in the index study. Clinical and demographic data for COVID-19 patients and unexposed subjects are reported in Table 1.

**Table 1:** Demographics and clinical features of subjects enrolled in the study.

	COVID-19 patients n = 32	Unexposed subjects n = 53	p value
Age, mean years (SD, range)	48.4 (13.7, 24–72)	43.7 (12.6, 25–64)	0.10*
Gender, n (%) males	22 (68.7)	23 (43.4)	0.03**
**Ethnicity, n (%)**			< 0.0001**
Caucasian	16 (50.0)	53 (100)	
Latin-American	9 (28.1)	0 (0)	
Indian	6 (18.7)	0 (0)	
African	1 (3.1)	0 (0)	
Body mass index (Kg/m^2^), mean (SD, range)	26.9 (4.5, 20.2–38.5)	23.9 (4.3, 17–37.1)	0.002*
**Comorbidities, n (%)**			
Systemic hypertension	8 (25.0)	9 (16.9)	0.41**
Diabetes	5 (15.6)	2 (3.8)	0.09**
HIV	1 (3.1)	0 (0.0)	0.37**
TB	1 (3.1)	0 (0.0)	0.37**
Alcohol consumption	7 (21.8)	19 (35.8)	0.22**
Smoking	8 (25)	15 (28.3)	0.80**
Dyslipidemia	4 (12.5)	5 (9.4)	0.72**
Coronary disease/stroke	1 (3.1)	0 (0.0)	0.37**
COVID-19 severity, n (%) severe	11 (34.4)	NA	NA

*COVID-19* coronavirus disease 19, *n* number, *SD* standard deviation, *NA* not available, *HIV* human immunodeficiency virus, *TB* tuberculosis.

*t-test.

**Fisher’s exact test.

All out of 32 subjects who had COVID-19 showed normal inflammatory markers with normal CRP and D-dimer levels at follow-up examination. Detailed values and comparisons between baseline and follow-up values of laboratory parameters in COVID-19 subjects are reported in Table 2.

**Table 2:** Laboratory parameters* in COVID-19 patients enrolled in the study (N = 32).

Parameter (units)	Baseline (acute COVID-19) Mean (SD, range)	Follow-up (post COVID-19) Mean (SD, range)	p**
HTC (%)	39.34 (5.52, 28 – 48)	42.4 (4.1, 33 – 49)	0.0003
WBC (× 10^6^/L)	6372.8 (2232.9, 1910–13,340)	7498.7 (1800.1, 4350–10,780)	0.006
Neutrophils (× 10^6^/L)	3723.1 (1798.7, 650–8730)	4331. 8 (1521.2, 1690–7130)	0.1
Lymphocytes (× 10^6^/L)	1874.7 (659.9, 770–3450)	2454.4 (713.8, 1340–4490)	< 0.0001
Platelets (× 10^9^/L)	312.3 (135.9, 113–673)	248 (54.4, 111–336)	0.01
PT (ratio)	1.2 (0.13, 0.97–1.61)	1.06 (0.1, 0.91–1.44)	< 0.0001
PTT (ratio)	1.16 (0.14, 0.9–1.47)	0.94 (0.1, 0.71–1.19)	< 0.0001
Fibrinogen (mg/dL)	536.5 (119.0, 320–701)	425.6 (108, 193–701)	0.003
D-Dimer (μg/L)	673.6 (501.5, 200–2279)	365.6 (177.8, 199–803)	0.003
CRP (mg/L)	21.8 (33.3, 1–142)	2.3 (1.5, 0.3–5)	0.002
Creatinine (mg/dL)	0.82 (0.18, 0.44–1.2)	0.84 (0.2, 0.39–1.26)	0.15

*COVID-19* Coronavirus disease 2019, *SD* standard deviation, *HTC* hematocrit, *WBC* white blood cells, *PT* prothrombin time, *PTT* partial thromboplastin time, *CRP* C-reactive protein, *LDH* lactic acid dehydrogenase, *CK* creatine kinase.

*The closest available within 3 days prior to the fundus examination at baseline and the same day of fundus photos at follow-up.

**T test (paired).

### Retinal findings and vessels analysis

At baseline examination eyes of COVID-19 patients had a series of retinal alterations including: retinal haemorrhages, cotton wool spots, veins dilation, vessels tortuosity, drusen (all features graded as present/absent by the retina specialist on fundus photography). At follow-up examination all the alterations except for drusen had significantly reduced compared to baseline. Details on single alterations prevalence and comparisons between baseline and follow-up are reported in Table 3.

**Table 3:** Retinal findings in subjects included in the study at baseline and follow-up.

Eyes N = 59 Patients N = 32	Baseline (acute COVID-19)	6 months F-UP (post COVID-19)	p***
Retinal hemorrhages Eyes (percentage)	12 (20.3)	1 (1.7)	0.0009
Cotton wool spots Eyes (percentage)	2 (3.4)	0 (0)	0.5
Drusen Eyes (percentage)	8 (13.6)	8 (13.6)	1
Dilated veins* Eyes (percentage)	12 (20.3)	4 (6.8)	0.007
Tortuous vessels* Eyes (percentage)	10 (16.9)	4 (6.8)	0.03
Mean artery diameter** µm (SD, range)	97.5 (10.9, 59.3–120.9)	92.2 (11.4, 55.4–114.7)	< 0.0001
Mean vein diameter** µm (SD, range)	133.1 (19.3, 98.9–201.34)	124.6 (16.1, 89.7–162.5)	< 0.0001

*COVID-19* Coronavirus disease 2019, *SD* standard deviation.

*According to qualitative evaluation performed by the ophthalmologists.

**Measured using the semi-automatic computer assisted method.

***McNemar Test for qualitative variables, linear mixed model for repeated measurements accounting for nesting of eyes within subjects for continuous variables.

**Figure 1:**
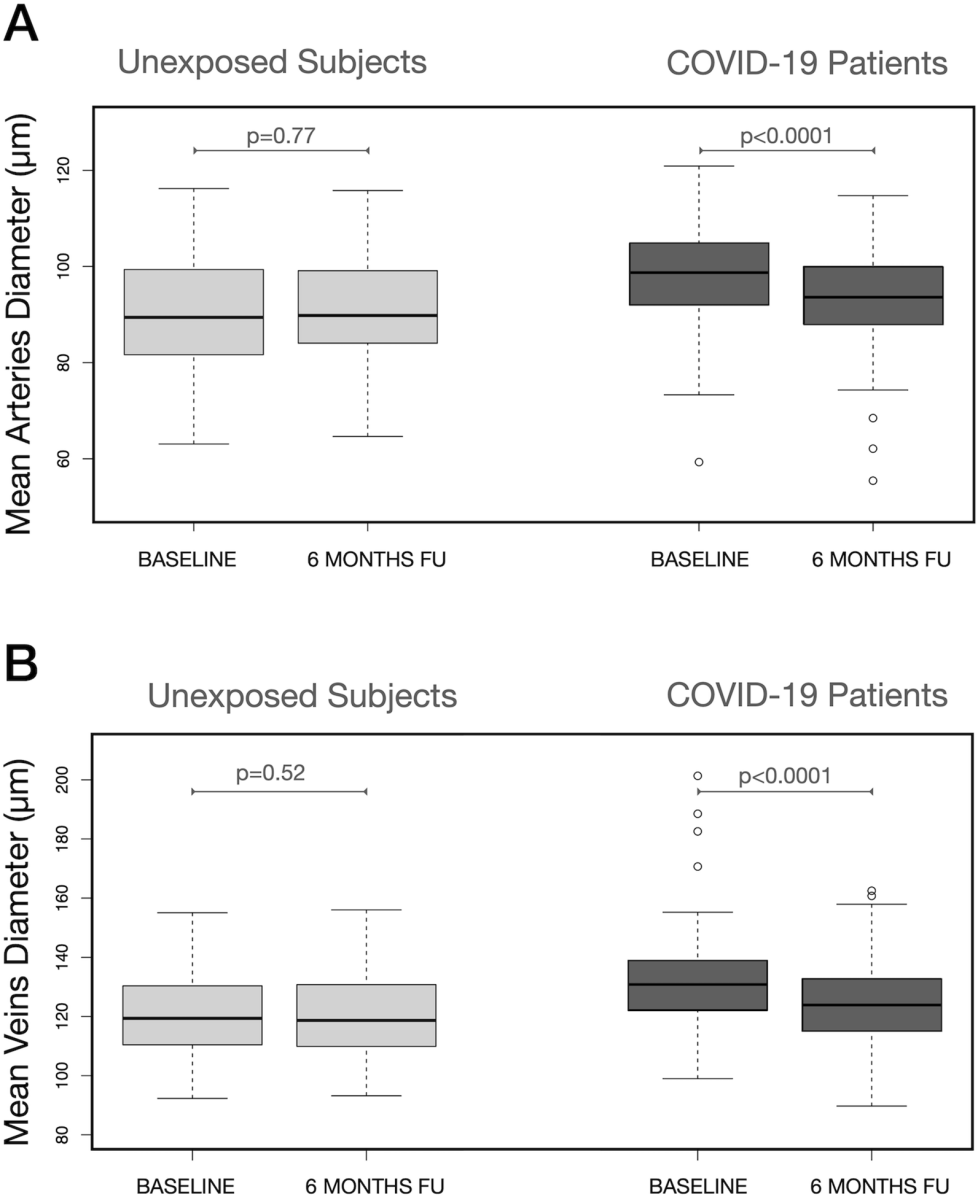
Mean Arteries diameter (MAD) and mean veins diameter (MVD) in COVID-19 patients and subjects unexposed to the SARS-CoV-2 virus at baseline and 6 months later: within groups comparison. Arteries (**A**) and veins (**B**) did not change in diameter from baseline to follow-up in subjects unexposed to the virus. By contrast both MAD (**A**) and MVD (**B**) significantly decreased from baseline to 6 months follow-up in patients who suffered from COVID-19. Reported p values were obtained by linear mixed model for repeated measurements accounting for nesting of eyes within subjects.

**Figure 2:**
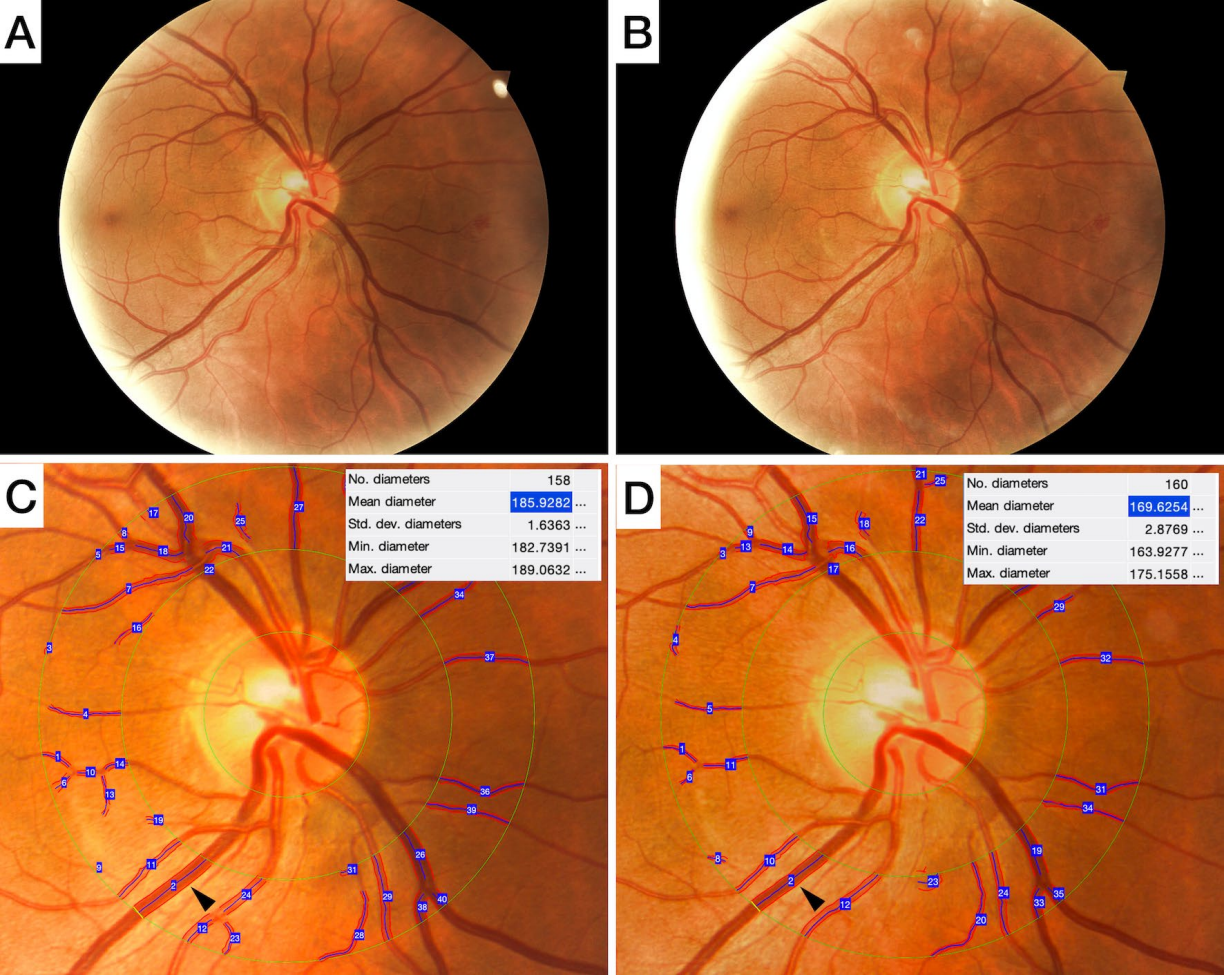
Fundus photographs of a COVID-19 patient during the acute phase of the disease and at 6 months follow-up. At baseline examination (**A**), during the acute phase of the disease retinal vessels, particularly veins, appear larger in diameter compared to those seen in the fundus photograph collected 6 months later (**B**). The difference is highlighted by the semi-automatic analysis of the vessels diameter in the circumpapillary area (**C,D**). In particular, the measurement box shows details of one of the four major veins (black arrow) whose diameter reduces from 185.9 µm at baseline to 169.6 µm 6 months later.

The semi-automatic analysis revealed that MAD significantly decreased in COVID-19 patients from 97.5 ± 10.9 µm at baseline to 92.2 ± 11.4 µm at 6 months follow-up (p < 0.0001). This did not happen in unexposed subjects in which no significant change was observed (from 90.6 ± 11.4 µm to 90.2 ± 11.6 µm, p = 0.77) (Table 3, Fig. 1A). Similarly, MVD significantly decreased from 133.1 ± 19.3 µm at baseline to 124.6 ± 16.1 µm at follow-up in COVID-19 patients (p < 0.0001) and did not change in unexposed subjects (from 120.4 ± 13.3 µm to 120.6 ± 13.1 µm, p = 0.52) (Table 3, Fig. 1B). An example of a comparison between fundus photos collected at baseline and 6 months later showing reduction in retinal veins diameter in a COVID-19 patient is reported in Fig. 2.

**Figure 3:**
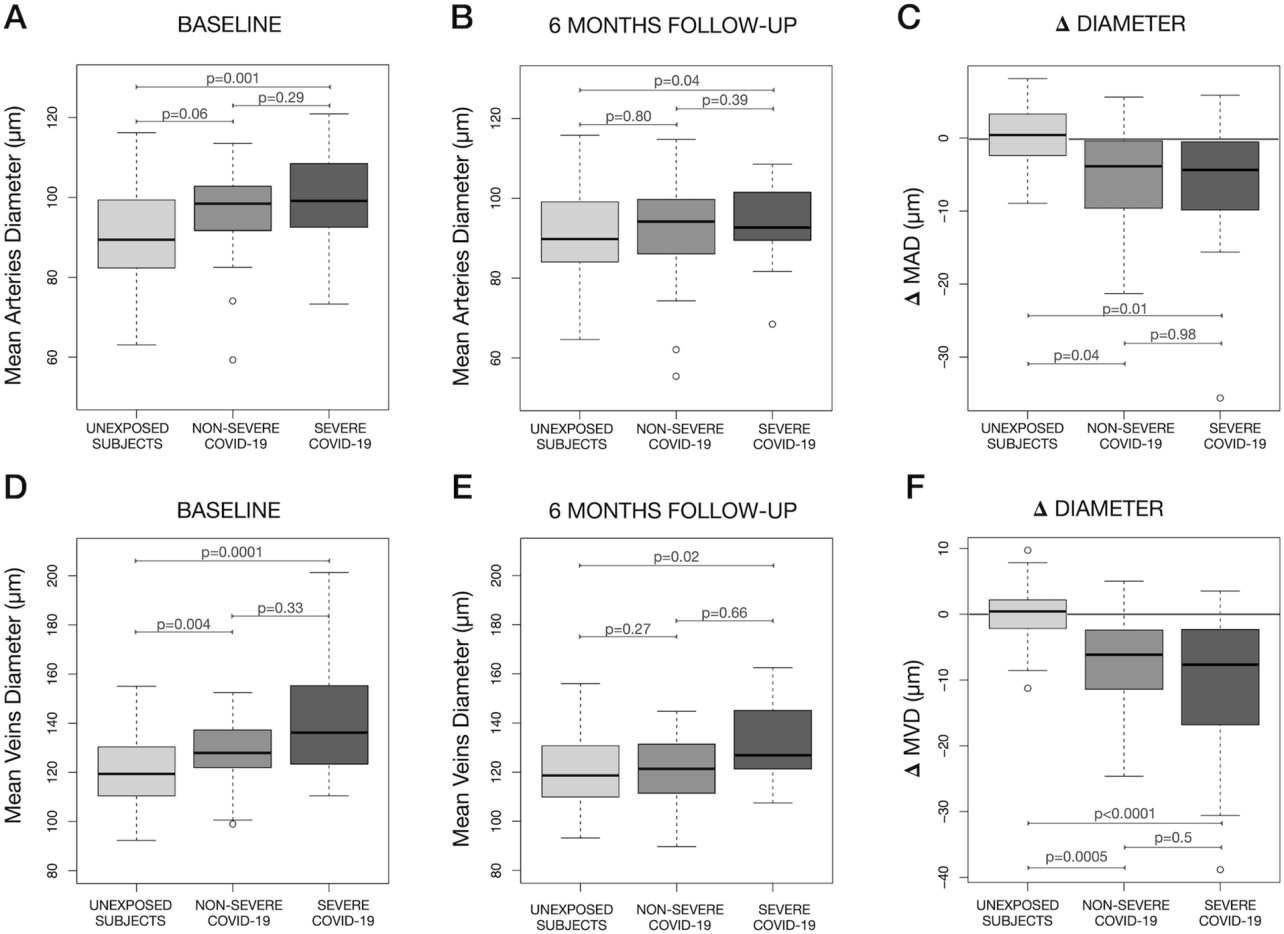
Comparisons in mean arteries diameter (MAD), mean veins diameter (MVD) at baseline and 6 months later and difference from baseline to follow-up (Δ Diameter) between severe COVID-19 patients, non-severe COVID-19 and subjects unexposed to the SARS-CoV-2 virus. At baseline MAD was higher in COVID-19 patients with a significant difference between severe COVID-19 and subjects unexposed to the virus (**A**). Six months later (**B**) MAD had reduced in both non-severe and severe COVID-19 patients but the arteries remained significantly thicker in severe COVID-19 compared to unexposed subjects. The reduction (Δ Diameter) was significantly higher in both severe and non-severe patients compared to controls. However the amount of change did not vary between severe and non-severe patients (**C**). At baseline MVD was significantly higher in both severe and non-severe patients compared to unexposed subjects (**D**). Six months later the difference remained significant only for severe cases (E). Similarly to what happened with MAD, the reduction (Δ Diameter) was significantly higher both in severe and non-severe patients compared to controls. However the amount of change did not vary between severe and non-severe patients (**F**). Comparisons are averaged through a multiple linear regression analysis over the levels of age, gender, ethnicity, body mass index, systemic hypertension, diabetes, smoking, alcohol consumption, dyslipidemia and history of coronary disease/stroke. p values for contrasts are also adjusted with a Bonferroni correction for 3 tests.

Multiple linear regression analysis accounting for the effect of covariates and using unexposed subjects as reference showed that both non-severe and severe COVID-19 patients had a higher MAD and MVD compared to unexposed subjects at baseline examination (Fig. 3A,D). The same analysis revealed that such difference was no more present in non-severe cases at follow-up, but both MAD and MVD remained significantly higher in severe COVID-19 patients compared to unexposed subjects (Fig. 3B,E).

After accounting for the effect of covariates both severe and non-severe COVID-19 showed a significant decrease in MAD and MVD from baseline to follow-up compared to unexposed subjects. No significant difference in the diameter change was found between severe and non-severe COVID-19 patients (Fig. 3C,F).

## Discussion

In this longitudinal study we analysed the retina and its vasculature in patients with COVID-19 during the acute phase of the disease and 6 months later. We found that most of the retinal vasculature alterations characterizing acute COVID-19 regress with time, but patients who suffer from severe COVID-19 may have long lasting retinal vessels dilation persisting at least 6 months after complete resolution of the disease.

There is increasing evidence of a damage induced by COVID-19 to the vascular system^17,18^, including the vessel supporting the ocular district^19^. Coagulation disorders are also common in COVID-19 patients, and both hospitalized and non-hospitalized subjects are at high risk for venous thromboembolism^20^. In addition, endotheliopathy due to direct endothelial infection with SARS-CoV-2 and the indirect damage caused by inflammation have been recognized in COVID-19^21,22^. The retinal vasculature is not different from that of other body districts and shares the same exposure to thromboembolic complications^23^. In fact multiple reports have highlighted signs of vascular disturbances in the retina of COVID-19 patients^7,8,24,25^.

Recently, we analyzed the retinal vessels of patients with acute COVID-19 and, besides non-specific signs of retinal microcirculation impairment like intraretinal hemorrhages and cotton wool spots, we found that both retinal arteries and veins were significantly dilated compared to those of subjects unexposed to the virus^6^. These findings had two possible explanations: a direct structural damage to the vessels walls caused by the virus^19^ or a transient modification induced by the cytokine storm^26^ typical of COVID-19^2,27^. In the current study we found that most of the retinal and vascular alterations of the acute phase regressed with time.

All clinically detectable retinal changes found at baseline, apart from drusen, significantly decreased at 6 months in our population. This suggests that haemorrhages and cotton wools, which have commonly been reported in COVID-19^6,28^, along with other thrombotic events to the retinal vasculature^7,8^, are likely related to an impairment to the retinal microcirculation^28^ induced by the transient hypercoagulability^20^ and the endothelial disfunction^29^ typical of the acute phase of the disease. Such damage to the microcirculation may persist at a subclinical level according to optical coherence tomography studies which demonstrated a decreased vascular density in the macular^10^ and peripapillary area^12^ of post-COVID-19 patients.

Retinal arteries diameter was increased in patients with acute COVID-19 compared to subjects unexposed to the virus and the difference in arteries diameter was statistically significant between severe cases and unexposed subjects^6^. Six months later the arteries of post-COVID patients had significantly decreased in diameter while those of unexposed subjects remained unchanged. This reduction in size suggests an at least partial reversibility of the arteries dilation induced by the acute phase of the disease. However, despite the dilation regression, the arteries of patients who had suffered from severe COVID-19 remained thicker at 6 months compared to those of unexposed subjects demonstrating a partial long lasting alteration.

In acute COVID-19 retinal veins dilation was significantly more pronounced in patients with severe disease compared to those with a non-severe clinical picture^6^. Interestingly, we found that six months after the disease resolution, retinal veins diameter had significantly decreased compared to baseline by a similar amount in both severe and non-severe COVID-19 patients. As a result, retinal veins returned similar to those of unexposed subjects only in patients who had non-severe COVID-19 while they remained significantly dilated in those who had severe disease. This difference, along with that found for arteries in severe cases, highlights that not all COVID-19 related alterations to the retina resolve after the acute phase of the disease.

The endothelial cells of both retinal arteries and veins express inflammatory cytokines receptors and the vessels can dilate in response to an inflammatory stimulus^26^. At baseline, during the acute phase of the disease, all our COVID-19 patients had important signs of systemic inflammation, while at 6 months all inflammatory and coagulation indexes had normalized. Similarly, both arteries and veins were dilated at baseline and decreased in diameter at six months. This change could hence be related to the inflammatory status of the patients. By contrast, retinal vessels in severe COVID-19 patients remained partially dilated at 6 months compared to those of non-severe patients and unexposed subjects. This difference in size could represent the amount of dilation caused by a structural and likely irreversible damage^18^. In fact, the endothelial activation in COVID-19 correlates with diseases severity^29^ and chronic vessels alterations have been documented in patients with severe COVID-19^30^. To further support this theory, recent pathology study on post-mortem eyes from patients who died from COVID-19 reported the presence of retinal vessels walls remodelling^19^.

Funduscopic examination provides a unique opportunity to analyse arteries and veins in vivo and it is commonly used to screen and grade the effects of systemic diseases on vessels walls^31,32^. Our study does not allow to clearly establish a correlation between the changes we identified in retinal vasculature and the increased risk for thrombotic events known to characterize COVID-19 patients. However, the acute and long lasting modifications to retinal vessels that we were able to observe in COVID-19 could easily be caused or accompanied by an endothelial damage^29^ and facilitate blood stasis^19^, two of the three factors of the Virchow’s triad, favoring cloths formation^33^. Further studies are needed to investigate the actual correlation between retinal alterations and thrombotic risk in COVID-19 patients.

We wish to acknowledge the limitations of our study. Many of the subjects included in the original cross-sectional analysis were not available at follow-up. We tried to partially overcome the drop by including two eyes from each subject when possible and accounting for this in the statistical analysis. Despite this, the absolute number of subjects included in the analysis remained limited and our results should be interpreted with caution, especially those stratifying COVID-19 patients according to disease severity. The SERPICO-19 study included only hospitalized patients with COVID-19, excluding asymptomatic subjects or those with mild symptoms. For this reason, our findings do not necessarily apply to all SARS-CoV-2 infected people. Baseline images were collected at the bedside with a fundus camera that could be easily moved around and that allowed a quick acquisition of the pictures. This was due to contingency of the moment with patients admitted to the hospital in strict droplet precaution. Indeed, wide field fundus images would likely provide more data, but their acquisition was not compatible with our pandemic setting. COVID-19 patients and unexposed subjects differed for many clinical and demographic characteristics with a potential impact on retinal vessels size. We tried to limit the effect of these differences on our results accounting for them in the statistical analysis thank to proper corrections. Finally, we only performed the retinal vessels measurements at two timepoints, during acute COVID-19 and 6 months later. Having multiple consecutive evaluations of the vessels’ diameter, especially one before the infection, would provide more data on the diameters’ variations overtime and their possible correlation with the inflammatory status of the patient.

To conclude, COVID-19 can induce important changes to the retinal vasculature during the acute phase of the disease, including microvascular infarcts and major arteries and veins dilation. Most of these alterations disappear six months after the disease resolution suggesting a possible correlation with the generalized inflammatory and pro-coagulant status typical of acute COVID-19. Patients who suffered from severe COVID-19 show persistent dilation of retinal vessels after the normalization of their systemic inflammatory parameters. These long lasting alterations likely reflect an irreversible structural damage. While transient vessels diameter modification could be explored as a possible biomarker for systemic inflammation, long lasting vascular damage in severe COVID-19 should be further investigated for its possible effects on tissues perfusion and function.

## Supplementary Information

Supplementary Information.

## Data Availability

The datasets generated during and/or analysed during the current study are available from the corresponding author on reasonable request.
