# Supplementary material for: Retinal vessels modifications in acute and post-COVID-19
Source: Sci Rep. 2021 Sep 29;11:19373. doi: 10.1038/s41598-021-98873-1 (PMC8481283; doi:10.1038/s41598-021-98873-1)
Supplement: Supplementary file 1 — Supplementary Information. [file 41598_2021_98873_MOESM1_ESM.docx]

Supplement Material 1

The diagram displays the recruitment process for the follow-up analysis of the SERPICO-19 study.
